# A head and neck treatment planning strategy for a CBCT‐guided ring‐gantry online adaptive radiotherapy system

**DOI:** 10.1002/acm2.14134

**Published:** 2023-08-24

**Authors:** Nour Nasser, George Q. Yang, Jihye Koo, Mark Bowers, Kevin Greco, Vladimir Feygelman, Eduardo G. Moros, Jimmy J. Caudell, Gage Redler

**Affiliations:** ^1^ Department of Radiation Oncology Moffitt Cancer Center Tampa Florida USA; ^2^ Department of Physics University of South Florida Tampa Florida USA

**Keywords:** Head and neck cancer, online adaptive radiotherapy, treatment planning

## Abstract

**Purpose:**

A planning strategy was developed and the utility of online‐adaptation with the Ethos CBCT‐guided ring‐gantry adaptive radiotherapy (ART) system was evaluated using retrospective data from Head‐and‐neck (H&N) patients that required clinical offline adaptation during treatment.

**Methods:**

Clinical data were used to re‐plan 20 H&N patients (10 sequential boost (SEQ) with separate base and boost plans plus 10 simultaneous integrated boost (SIB)). An optimal approach, robust to online adaptation, for Ethos‐initial plans using clinical goal prioritization was developed. Anatomically‐derived isodose‐shaping helper structures, air‐density override, goals for controlling hotspot location(s), and plan normalization were investigated. Online adaptation was simulated using clinical offline adaptive simulation‐CTs to represent an on‐treatment CBCT. Dosimetric comparisons were based on institutional guidelines for Clinical‐initial versus Ethos‐initial plans and Ethos‐scheduled versus Ethos‐adapted plans. Timing for five components of the online adaptive workflow was analyzed.

**Results:**

The Ethos H&N planning approach generated Ethos‐initial SEQ plans with clinically comparable PTV coverage (average PTV_High_ V_100%_ = 98.3%, D_min,0.03cc_ = 97.9% and D_0.03cc_ = 105.5%) and OAR sparing. However, Ethos‐initial SIB plans were clinically inferior (average PTV_High_ V_100%_ = 96.4%, D_min,0.03cc_ = 93.7%, D_0.03cc_ = 110.6%). Fixed‐field IMRT was superior to VMAT for 93.3% of plans. Online adaptation succeeded in achieving conformal coverage to the new anatomy in both SEQ and SIB plans that was even superior to that achieved in the initial plans (which was due to the changes in anatomy that simplified the optimization). The average adaptive workflow duration for SIB, SEQ base and SEQ boost was 30:14, 22.56, and 14:03 (min: sec), respectively.

**Conclusions:**

With an optimal planning approach, Ethos efficiently auto‐generated dosimetrically comparable and clinically acceptable initial SEQ plans for H&N patients. Initial SIB plans were inferior and clinically unacceptable, but adapted SIB plans became clinically acceptable. Online adapted plans optimized dose to new anatomy and maintained target coverage/homogeneity with improved OAR sparing in a time‐efficient manner.

## 1 INTRODUCTION

Conventional radiotherapy (RT) consists of generating an initial treatment plan based on a pre‐treatment CT and associated relevant anatomy/disease delineated by the physician, and subsequently treating patients with this initial treatment plan throughout the entire treatment course, usually lasting a few weeks. This is based on the assumption that the initial patient model/plan remains applicable for the full treatment course. However, daily changes in patient positioning and/or patient anatomy (e.g., weight, tumor size, organ filling, etc.) can lead to uncertainties and even result in missing the tumor and/or depositing additional doses in surrounding normal tissues potentially resulting in treatment failures and/or unwanted toxicities.^1^ Margins to account for these uncertainties are incorporated into the treatment planning process; however, this may limit the ability to deliver tumoricidal radiation doses without surrounding normal tissue toxicity.^2^, ^3^

The concept of adaptive radiotherapy (ART) was introduced in 1997, where initial treatment plans are adjusted accounting for variations throughout a treatment course.^4^ ART can be offline (conventional treatment planning process described above is repeated) or online (plan adaptation is performed with the patient on the table, prior to treatment delivery, based on anatomy of the day).^5^ Offline ART showed improvement in target coverage and organ‐at‐risk (OAR) sparing for several treatment sites such as prostate, head and neck (H&N), and lung.^6^, ^7^, ^8^ Similarly, Online ART (oART) has shown improvement in target coverage and OAR sparing for standard fractionation and stereotactic RT for H&N,^9^ abdomen,^10^, ^11^, ^12^ pelvic^13^, ^14^, ^15^ and, ultra‐central lung.^16^

The newly developed Ethos CBCT‐guided ring‐gantry oART system (Varian Medical Systems, Palo Alto, CA) is based on the Varian Halcyon treatment machine with a closed‐bore allowing faster four revolutions per minute (RPM) gantry rotation and a 6MV flattening filter free beam. The multi‐leaf collimators are dual‐stacked and staggered, providing 0.5 cm effective leaf width at the isocenter, without additional collimating jaws. Currently, Ethos uses iteratively reconstructed kV‐CBCT for image guidance.^17^ Ethos provides automated planning with the intelligent optimization engine (IOE) using physician clinical goals to automatically generate the optimization objective functions for the photon optimization (PO) algorithm.^18^ The IOE automatically creates helper structures (e.g., dose‐shaping rings for targets), objectives to control monitor units (MU), and objectives for dose fall off from targets to spare normal tissue.^18^ The IOE also creates cropped non‐overlapping structures for dose optimization to help achieve clinical goals based on assigned priority levels. There are four priority levels for clinical goals, from P1 (most important) to P4 (least important). Additionally, there is an “R” priority, which will only report a given dosimetric value for evaluation purposes. The IOE reassigns weights for the objectives to most closely achieve user‐provided clinical goals. Note, this is a novel automated planning paradigm that differs from traditional manual manipulation of optimization objective functions to indirectly achieve physician goals, as is done in most conventional treatment planning systems (TPSs). As such, one focus of this work was to develop/evaluate a planning strategy within this paradigm for H&N that would produce clinically acceptable initial plans and was robust enough to maintain plan quality during oART.

The online adaptive workflow in Ethos (v1.1) generates a synthetic CT for dose calculation using deformable image registration (DIR) of the initial planning CT to the CBCT of the day.^18^, ^19^ During the adaptive process, only certain auto‐segmented (via artificial intelligence (AI) models and/or DIR) structures are reviewed and edited when needed. This includes a subset of OARs called “influencers” that are used for structure‐guided DIR to generate remaining OARs and targets.^19^ Targets as well as OARs with high priority (P1 or P2) are reviewed and edited, while remaining structures are not visible or editable. Ethos generates a “scheduled plan” (initial plan re‐calculated on the anatomy of the day) and an “adapted plan” that has the same beam geometry as the initial plan but is re‐optimized to adapt the dose based on the anatomy of the day using the IOE with the same clinical goals as the initial plan.^19^

In this study, we evaluated the capabilities of Ethos for H&N online adaptive radiotherapy by developing an optimal strategy to generate clinically acceptable initial plans as well as efficiently/effectively provide online plan adaptation to improve overall treatment quality. Clinical data from previously treated H&N patients that received offline adaptation during their treatment course were used to provide extreme scenarios where real anatomical changes were appreciable enough to clinically justify an offline plan adaptation with complete replanning. These significant changes in patient anatomy were chosen to push the limits of the Ethos online adaptive system. This differentiates this work from many previous studies that evaluated this system on periodically sampled patient CBCTs, which understandably often included lesser anatomical perturbations.^1^, ^20^, ^21^, ^22^ Demonstrated success of such adaptation in this work will emphasize the robustness of the optimizer and its ability to account for not only minor but major changes. Use of cases with clinical offline adapted data also provided benchmark clinical dosimetry for adapted plans.

## 2 MATERIALS AND METHODS

### 2.1 Initial planning

For this study, retrospective clinical data, such as simulation‐CT and physician defined structures, were used to re‐plan, in Ethos, 20 previously treated H&N patients. In total, 30 H&N initial plans were created in Ethos for the 20 patients’ cohort: 10 simultaneous integrated boost (SIB) patients (10 plans) and 10 sequential boost (SEQ) patients (20 plans: 10 base and 10 boost plans). The distribution of these patients was: 60% Oropharynx, 15% Oral cavity and 5% of each hypopharynx, nasopharynx, larynx, maxillary sinus, and unknown primary. These patients were selected because they all required offline adaptation during their treatment course due to considerable anatomical changes. A summary of the initial planning and online adaptive process for H&N patients within Ethos highlighting what data were analyzed, is in Figure S1 and has generally been described previously in the literature.^1^, ^20^, ^23^ Manual contouring of GTV and CTV_Med/Low_ was performed by subspecialized H&N radiation oncologists where CTV_High_ = GTV + 5 mm and PTV_High/Med/Low_ = CTV_High/Med/Low_ + 3 mm. Following expansion of GTVs, clinical CTV_High_ was manually cropped from regions where physicians were confident that disease had not spread (e.g., bone, air, fascial planes, etc.) and PTVs were cropped 5 mm from the skin to allow for dose build‐up.

In this study, plans were stratified based on the boost being incorporated into all fractions of a given plan, which is the case for SIB patients, or the boost being treated in 10 fractions that do not include treatment of PTV_low_, which is the case for SEQ patients. Ten patients were planned based on SIB with different PTV dose levels. These were distributed as follows (PTV_High_/PTV_Med_/PTV_Low_ [Gy]): 70/‐/56 (*n* = 5), 66/‐/52.8 (*n* = 2), 69.3/‐/54.12 (*n* = 1), 70/63/56 (*n* = 1), 66/60/54 (*n* = 1). The remaining 10 patients were planned based on SEQ with the total (base plus boost plan) PTV dose levels as follows (PTV_High_/PTV_Med_/PTV_Low_ [Gy]): 66/‐/41.4 (*n* = 4), 66/59.4/41.4 (*n* = 3), 66/‐/40 (*n* = 1), 66/46/41.4 (*n* = 1), 66/(59.4 & 46)/41.4 (*n* = 1). Note that SEQ base plans had multiple PTV dose levels as well, similar to SIB plans.

For this work, an Ethos emulator software package was utilized (Ethos v1.1, Varian Medical Systems, Inc., Palo Alto, CA). The emulator provided the Ethos TPS for initial planning and capabilities to simulate the online adaptive workflow in silico without requiring a fully functioning clinical system. For initial plan generation, clinical structures (GTVs, CTVs, and OARs) were imported into a templated Ethos planning directive for efficiency and consistency. PTVs and CTVs were derived from GTVs using the above clinical margins. Similarly, planning organ at risk volumes (PRVs) were created using Ethos derivation so that they propagated appropriately when GTV/critical OARs were modified during adaptation. The nomenclature suggested by AAPM TG263 was used, where structure names ending in “_PRVxx” represented OAR expansions of xx millimeters.^24^

Specific institutional guidelines and associated clinical goals/priorities for the initial planning approach in Ethos are in Table 1. The general planning approach was as follows. PTV and critical OAR (spinal cord, brainstem) goals were assigned higher priority (P1–P2), and other OAR goals were assigned lower priority (P3–P4). To minimize clinical goals, only the most important goal for each OAR was used, and goals for OARs intersecting or very close to PTVs that may cause conflict and prevent the IOE from generating a clinically acceptable plan were excluded altogether (these were decided on a case‐by‐case basis by identifying regions within PTVs that had unacceptably high hot spots or appreciable under‐coverage and were in close proximity to OARs with conflicting objectives). For hotspot control, a goal of D_0.03cc_ ≤ 105% was used when planning, with 107% acceptable variation, which was based on what was found to be achievable for H&N plans using a similar dose calculation algorithm.^25^

**TABLE 1:** List of clinical goals and their corresponding priority for the initial planning approach in Ethos treatment management system based on institutional guidelines.

Structure name	Goal	Priority
PTV_High, Med, Low_	D_min,0.03cc_ ≥ 95 %	1
	V_100%_ ≥ 95 %	1
PTV_High, Med, Low_	D_0.03cc_ ≤ 105 % (107 %)^a^	2^b^
SpinalCord_PRV05	D_0.03cc_ < 50 Gy	2
	V_30Gy_ < 45%	3
	V_40Gy_ < 10%	3
	D_mean_ < 26 Gy	4
BrainStem_PRV03	D_0.03cc_ < 54 Gy	2
	D_2.7cc_ < 55 Gy	3
	D_0.9cc_ < 60 Gy	3
	D_mean_ < 36 Gy	4
Brain	V_50Gy_ < 10 %	4
Parotids	D_mean_ < 26 Gy	3^c^
Submandibular glands	D_mean_ < 39 Gy	4^c^
Oral Cavity	D_mean_ < 32 Gy	4^c^
Larynx	V_35Gy_ < 79 %	
	V_45Gy_ < 45 %	
	V_55Gy_ < 32 %	
	V_65Gy_ < 22 %	
	D_mean_ < 51 Gy	4^c^
Thyroid gland	D_mean_ < 40 Gy	4^c^
Pharynx constrictor superior (PCS)	D_mean_ < 54 Gy	4^c^
Pharynx constrictor middle (PCM)	D_mean_ < 54 Gy	4^c^
Pharynx constrictor inferior (PCI)	D_mean_ < 54 Gy	4^c^
	V_40Gy_ < 65 %	
	V_50Gy_ < 47 %	
	V_60Gy_ < 11 %	
Mandible	D_0.03cc_ ≤ 73.5 Gy	
	V_70Gy_ < 6.5 %	
	V_60Gy_ < 35 %	4^c^
	V_50Gy_ < 62 %	4^c^

*Note*: In general, PTVs and critical OAR goals are assigned higher priority (P1‐P2), and other OAR goals are assigned lower priority (P3‐P4). Numbers at the end of a structure name for PRVs represent the isotropic expansion in mm.

^a^ 105% used for planning and 107% (acceptable variation) used in boxplots.

^b^ Goals used when needed for PTV_Med, Low._

^c^ Goal excluded if too close or intersecting PTV.

**FIGURE 1:**
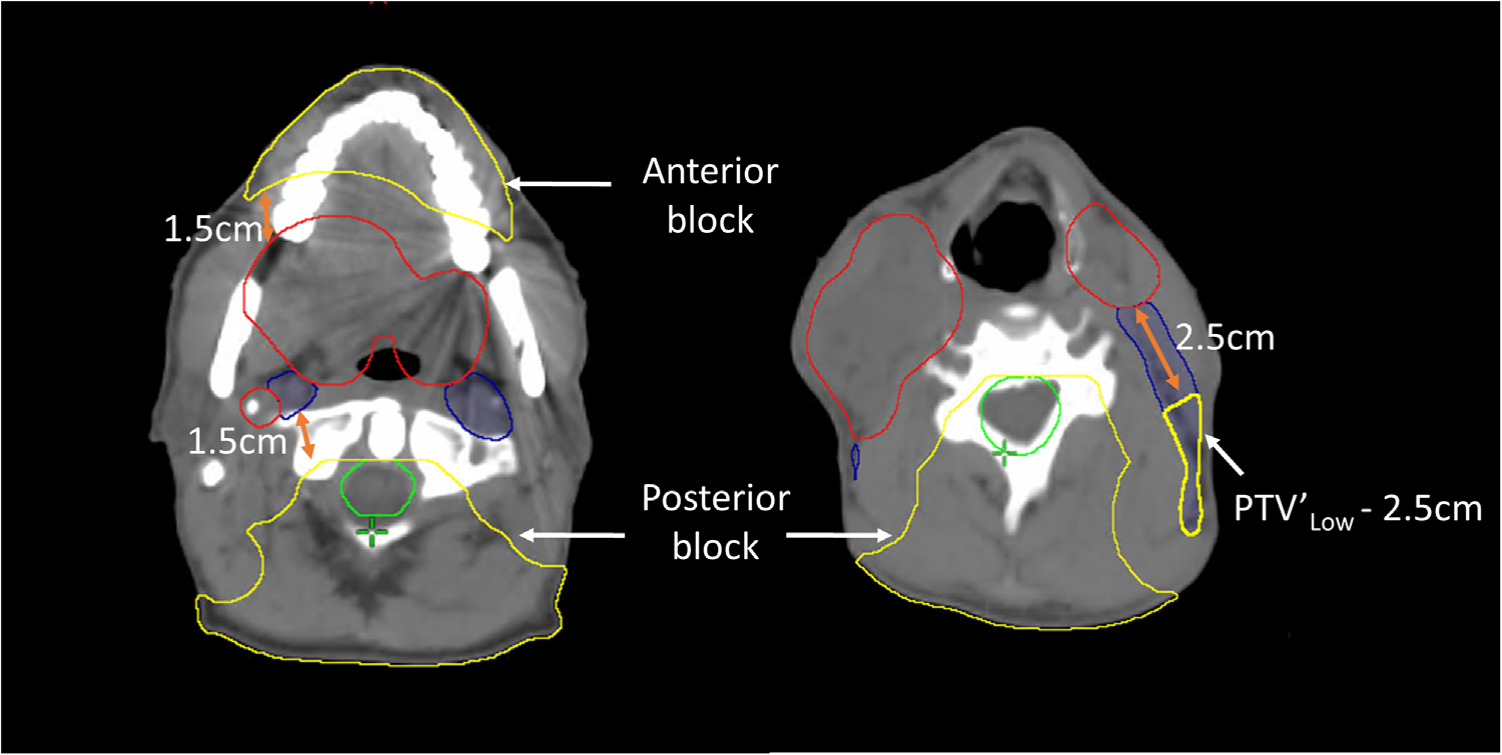
Illustration of the anatomically‐derived helper structures described in Table 2 for isodose shaping and hotspot control. PTV_High_ (red), PTV_Low_ (blue), union of brainstem and spinal cord PRVs (green), and anatomically‐derived helper structures (yellow).

Additional structures and goals were introduced to control undesired hotspots in PTV high, medium and low, such as V_105%_ ≤ 5% with intermediate priority, as well as additional goals (D_0.03cc_ ≤ 105% and V_105%_ ≤ 5%) to cropped structures of PTV_Med, Low_ defined as follow: PTV_Med_ and/or PTV_Low_ were cropped a certain distance from PTV_High_. This distance was determined based on the prescription dose to PTV_Med_ and/or PTV_Low_ relative to the prescribed dose to PTV_High_. This distance was either 1.5 or 2.5 cm away from PTV_High_ for prescribed doses of 90% or 80% relative to that of PTV_High_, respectively (Table 2). To limit hotspots outside of the targets, additional cropped body structures from all PTVs were created and assigned a goal of D_0.03cc_ < PTV_High_ prescription dose. Anatomically‐derived helper structures for isodose shaping, such as low dose in the posterior neck and oral cavity, were defined with lower priority goals (Table 2). For the posterior block, a margin around the union of the brainstem and the spinal cord was taken 9 cm laterally and posteriorly with the resulting structure then cropped 1.5 cm away from the target. For the anterior block, the oral cavity was cropped 1.5 cm away from the target. It is critical to note that the above helper structures must be anatomically derived (i.e., not edited manually as is common practice in more standard optimization‐based planning approaches) so that these helper structures adapt appropriately during the oART process (Figure 1).

**TABLE 2:** List of additional anatomically‐derived helper structures with their derivation, associated planning goals and priorities used in the ethos treatment management system for hotspot control and isodose shaping. “PTVs” is the combination of all targets.

Helper structure name	Helper structure function:	Helper structure derivation	Goal	Priority
PTV_High, Med, Low_	PTV undesired hotspot	NA	V_105%_≤5%	2‐3^a^
PTV’_Med_ −1.5 cm	PTV undesired hotspot	PTV_Med_—(PTV_High_ + *1.5* *cm*)	D_0.03cc_≤105%	2^a^
			V_105%_≤5%	2^a^
PTV’_Low_ −2.5 cm	PTV undesired hotspot	PTV_Low_—(PTV_High_ + *2.5* *cm*)	D_0.03cc_≤105%	2‐3^a^
			V_105%_≤5%	2‐3^a^
Body—PTVs	Normal tissue high dose	(Body—PTVs)	D_0.03cc_≤100%	3
Anterior block	Oral cavity undesired low dose	OralCavity—(PTVs + 1.5 cm)	V_35Gy_ < 10%	4^a^
Posterior block	Posterior neck undesired low dose	[(BrainStem U SpinalCord) + *m_1_* ‐ (PTVs + *m_2_*)] ∩ Body	D_0.03cc_≤50%	3
		*m_1_* = 9 cm left/right/posterior *m _2_* = 1.5 cm (isotropic)	D_mean_ < 25%	3

*Note*: U, +, ‐, and ∩ represent the union, expansion, subtraction, and intersection operations, respectively. PTV goals listed with a relative dose (e.g., 105%) is with respect to that particular PTV's prescription dose. If unspecified, expansions are assumed to be isotropic.

^a^ Goal used as needed.

In Ethos version 1.1, plan normalization must be set prior to plan generation. This was used sparingly to normalize to PTV_High_ D_min,0.03cc_ = 95% in cases that required this to ensure appropriate target coverage. Note, D_min,0.03cc_ refers to the minimum dose that covers all but 0.03cc of a given structure. However, the initial plan normalization was applied to subsequent adapted plans as well (unless a plan revision was created offline to remove plan normalization) and thus could lead to unwanted plan degradation, depending on anatomical changes during online adaptation. In technical structures, a separate module in Ethos, density correction with manual override of any desired area was possible. For a subset of cases with particularly complex targets, air in/near the PTV was manually contoured and overridden to water density in order to aid dose calculation and optimization.

In Ethos, once clinical goals have been defined, the user moves on to dose preview. Here, the IOE and PO generate preliminary dose calculation for a generic 9‐field beam geometry based on Fourier transform (FT) dose calculation (rather than final plan beam geometries and Acuros XB, respectively). This provides a rapid, albeit less accurate, dose calculation with the achieved dosimetric goals, isodose lines and dose‐volume histogram (DVH). In dose preview, fine tuning is available by re‐ordering clinical goals within each level, with real‐time dose calculation to observe interplay between different clinical goals. Once clinical goal prioritization was approved within dose preview, the IOE generated up to five candidate plans in plan preview (7/9/12 equally spaced fixed‐field IMRT and 2/3 full‐arc VMAT), from which the user may select the optimal initial plan and associated clinical goals for use in online plan adaptation.

Different planning approaches were tested on SIB plans due to the complexity of such plans (e.g., varying clinical goals and priorities, including/excluding goals for OARs intersecting PTVs, etc.). The approach that resulted in clinically acceptable SIB Ethos plans was used in this study to plan all patients. Ethos plans were assessed based on our institutional guidelines, since both clinical and Ethos plans were planned and approved based on these guidelines. To provide objective plan quality evaluation, an established plan quality metric (PQM) was used.^26^ This PQM is based on specific metrics such as DVH points with their corresponding score functions that translate the achieved goal to a numerical score. The score functions have a failure region with zero score, a transition region from the minimum acceptable achieved goal to the ideal goal with an increasing score from zero to maximum, and a region exceeding the ideal goal with a maximum score. The composite PQM (%), which represents the sum of all metric scores divided by the combined maximum possible, was calculated for Ethos‐initial and Clinical‐initial plans in PlanIQ (v.2.1, Sun Nuclear Corp, Melbourne, FL).

The version of Ethos used did not have tools to sum multiple plans (e.g., base plus boost composite dose) or compare multiple plans simultaneously; therefore, all SEQ plans (base and boost) were exported to Eclipse (Varian Medical Systems, Palo Alto, CA) where dosimetric data were analyzed. The Ethos plans were calculated using the Acuros XB linear Boltzmann transport equation (LBTE) solver, whereas the clinical plans were generated using Pinnacle (Philips Medical Systems, Fitchburg, WI, USA) collapsed cone (CC) superposition/convolution.

### 2.2 Online adaptation

Online Adaptive RT was simulated in Ethos for all patients using retrospective simulation‐CT, originally used clinically for offline adaptation, to represent the patient's online CBCT (referred to as “pseudo‐CBCT” in this work) at a time point during a treatment course where adaptation was required due to significant changes in patient anatomy. This clinical offline adaptive simulation‐CT was cropped craniocaudally to match the length of the expected CBCT (24.5 cm) based on maximum length in a single plan with single isocenter as well as laterally to place the “image acquisition isocenter” (set by the emulator as the center of the image dataset) in a location approximating the anatomical location of the isocenter from the initial Ethos plan (which was automatically placed at the centroid of the box encompassing all targets). This approximation of where the CBCT image acquisition center might be in a true clinical scenario is important since, for the Ethos adapted plans, the treatment isocenter will be defined as the image acquisition center. For Scheduled plans, a rigid shift will be determined to place the treatment isocenter at the center of the box surrounding the new targets, in order to approximately match the initial plan isocenter. Following “image acquisition” (i.e., loading of pseudo‐CBCT into the emulator), influencer structures such as parotids, mandible, and spinal canal were used for the structure‐guided DIR and deformable propagation of target(s)/OARs from initial simulation‐CT to pseudo‐CBCT. These influencer structures were auto‐generated (initial global DIR) for review/edit followed by auto‐generation of targets as well as OARs with priority 1 or 2 (via influencer‐guided DIR) for physician review/edit. Structures with lower priority (3 and 4) were not available to review/edit during the oART workflow. In this H&N adaptive workflow (Ethos v1.1), structure auto‐segmentation is not AI‐based, unlike other anatomical sites such as the abdomen and pelvis. Only underived targets such as GTV and/or CTV_Med/Low_ that were used to derive PTV were auto‐generated for review/editing. PTVs and PRVs were adaptively auto‐expanded, as specified in the initial plan derivation rules, based on relevant OAR/GTV/CTV structures of the day.

The adapted plan utilizes the same beam geometry selected for the initial plan but beam intensities are re‐optimized based on initial clinical goals applied to structures and scan of the day. The scheduled plan is the initial plan re‐calculated, without beam intensity changes, on the structures and scan of the day. The “reference” (initial plan on initial scan/contours), “scheduled”, and “adapted” Ethos plans are calculated and achieved clinical goals; isodose lines/color wash and DVH are available for evaluation. Dosimetric comparison of clinical‐initial versus Ethos‐initial plans and Ethos‐scheduled versus Ethos‐adapted (online) plans were performed based on institutional guidelines. Additionally, the composite PQM was calculated for the offline clinical‐adapted and online Ethos‐adapted plans. For SEQ plans, offline clinical‐adapted plans were only available for the boost phase, therefore this PQM comparison was only evaluated for offline clinical and online Ethos adapted boost plans (see Table 3). To evaluate the logistical efficiency of online adaptation in these cases with Ethos, timing data for the five different components, from influencers to plan generation, of the adaptive workflow were collected as well.

**TABLE 3:** Composite PQM (%) statistics for the initial and adapted Ethos and clinical plans.

Composite PQM (%)								
	SIB plans				SEQ plans			
	Initial plan		Adapted plan		Composite initial plan		Adapted boost plan	
	Ethos	Clinical	Ethos	Clinical	Ethos	Clinical	Ethos	Clinical
Average	57.2	60.1	61.9	63.5	71.2	75.9	73.0	79.5
σ	9.3	11.5	8.6	7.4	8.8	7.0	9.0	9.9
Min	46.6	39.9	50.3	50.6	56.2	63.3	57.4	60.8
Max	76.7	78.2	75.5	72.3	83.7	85.6	85.9	93.4

All data gathering and statistical tests were performed on Microsoft Excel (Microsoft, Redmond, WA). Dosimetric indices for Ethos and clinical plans were compared via two‐sided student paired t‐test (*p* < 0.05 considered significant).

## 3 RESULTS

### 3.1 Initial planning

In Ethos (v1.1), fixed‐field IMRT plans consistently showed higher plan quality (i.e., better and more consistently achieved PTV goals), resulting in the selection of fixed‐field IMRT over VMAT plans 90.0% of the time (33.3%, 36.7%, 20.0%, 3.3%, 6.7% for 7‐, 9‐, 12‐field IMRT and 2‐, 3‐full arc VMAT, respectively). Average modulation factors (plan MU per PTV_High_ prescribed cGy) for Ethos SIB, SEQ base, and SEQ boost plan were 8.4 ± 1.7, 7.6 ± 0.7, and 4.4 ± 1.2, respectively.

Overriding air density to water within targets in Ethos plans was used to help decrease undesired hotspots when the optimizer was working to achieve homogeneous coverage within targets containing air. This approach was used sparingly and only after all other approaches failed to achieve clinical goals, which resulted in application to 30.0% of plans: 6/10 SIB, 2/10 SEQ base, 1/10 SEQ boost. Normalization to PTV_High_ was often necessary to achieve the D_min,0.03cc_ goal in initial plans and it was used for 75% of the plans.

**FIGURE 2:**
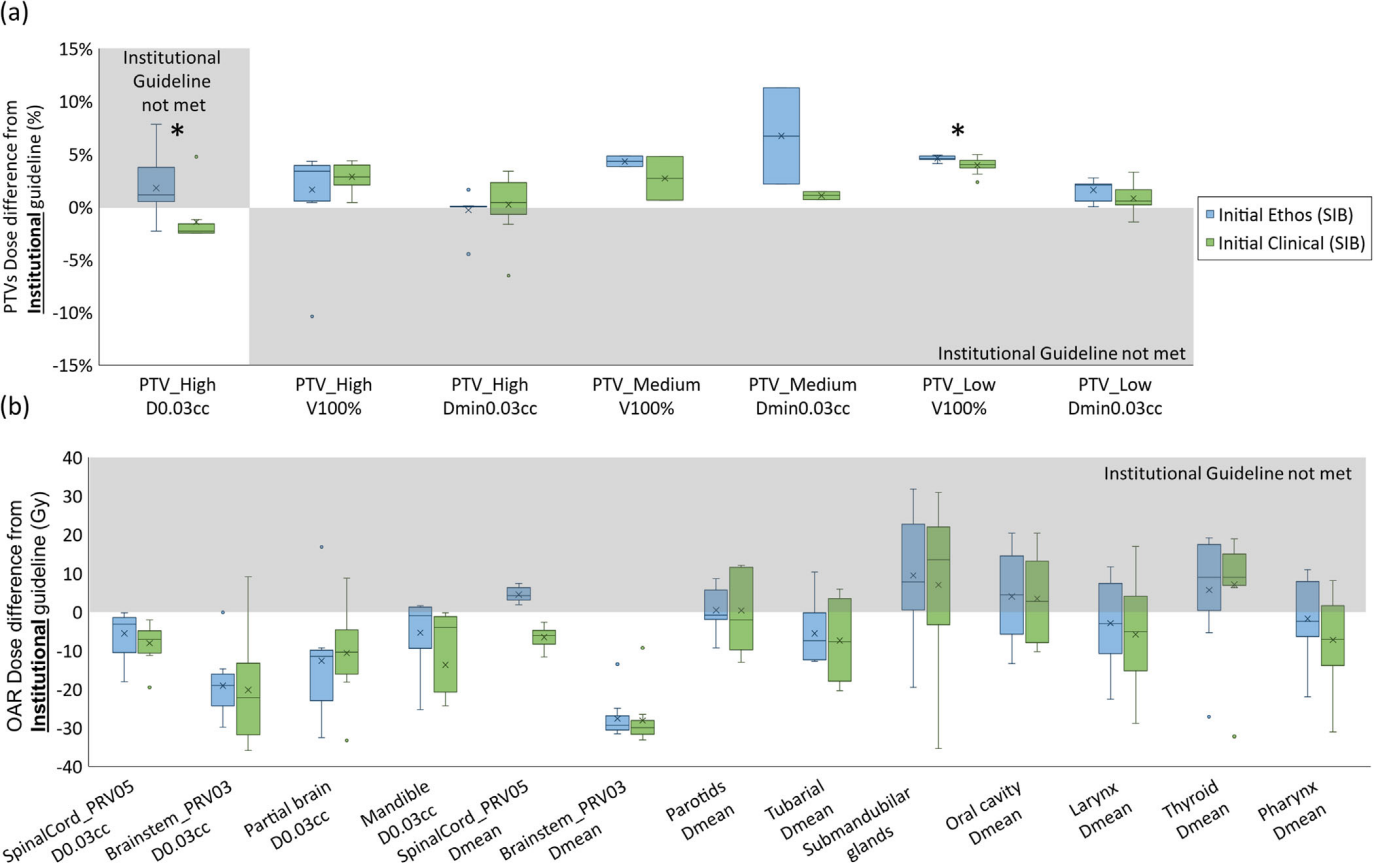
SIB initial plan dosimetric evaluation. Distributions of (a) PTV (b) OAR dose differences between achieved values and institutional guidelines for Ethos (blue) and clinical (green) initial plans. The shaded areas indicate that a goal was not met. *Statistically significant difference (*p* < 0.05).

Figure 2 shows the difference from institutional guidelines of (a) PTV and (b) OAR achieved goals for Ethos and clinical initial SIB plans. All boxplots in this study were based on the difference between the achieved goal in Ethos/clinical plans and institutional clinical goals with one acceptable variation: D_0.03cc_ ≤107% instead of ≤105%. This allowed variation was associated with plan quality changes observed when moving from collapsed cone calculation approaches to more rigorous algorithms.^25^ For most goals, the PTV and OAR dose differences from institutional guidelines in both Ethos‐ and clinical‐initial SIB plans were comparable. However, PTV_High_ achieved goals in Ethos were clinically unacceptable in some cases. PTV_High_ coverage in Ethos was slightly below clinical plans with significantly higher hotspots: average V_100%_ = 96.4%/97.1%, D_min,0.03cc_ = 93.7%/96.2%, and D_0.03cc =_ 109.26%/105.4% in Ethos and clinical plans, respectively. Ethos initial plan global hotspot was successfully placed within the targets as verified by ensuring a lower normal tissue (Body—PTVs) D_0.03cc_ with an average of 107.44% in all plans except for one plan with 3.5 cGy higher hotspot outside of the PTV_High_. OARs in both plans were comparable with a trend towards slightly higher doses in Ethos plans, but with no statistical significance. The difference was statistically significant (favorable for clinical versus Ethos plans) for PTV_High_ D_0.03cc_ (*p* = 0.002) and PTV_Low_ V_100%_ (*p* = 0.025). The statistically significant difference seen for PTV_Low_ V_100%_ (as well as the other trends above that were not statistically significant) was mainly due to the automated planning paradigm in Ethos. The traditional approaches in conventional planning (i.e., that used for clinical plan generation) that allow for manual manipulation of small regions of unwanted high or low doses is no longer possible within the Ethos planning paradigm, nor is it practical within the online adaptive setting. This results in slightly diminished plan results for metrics that are governed by changes in dose to smaller volumes.

**FIGURE 3:**
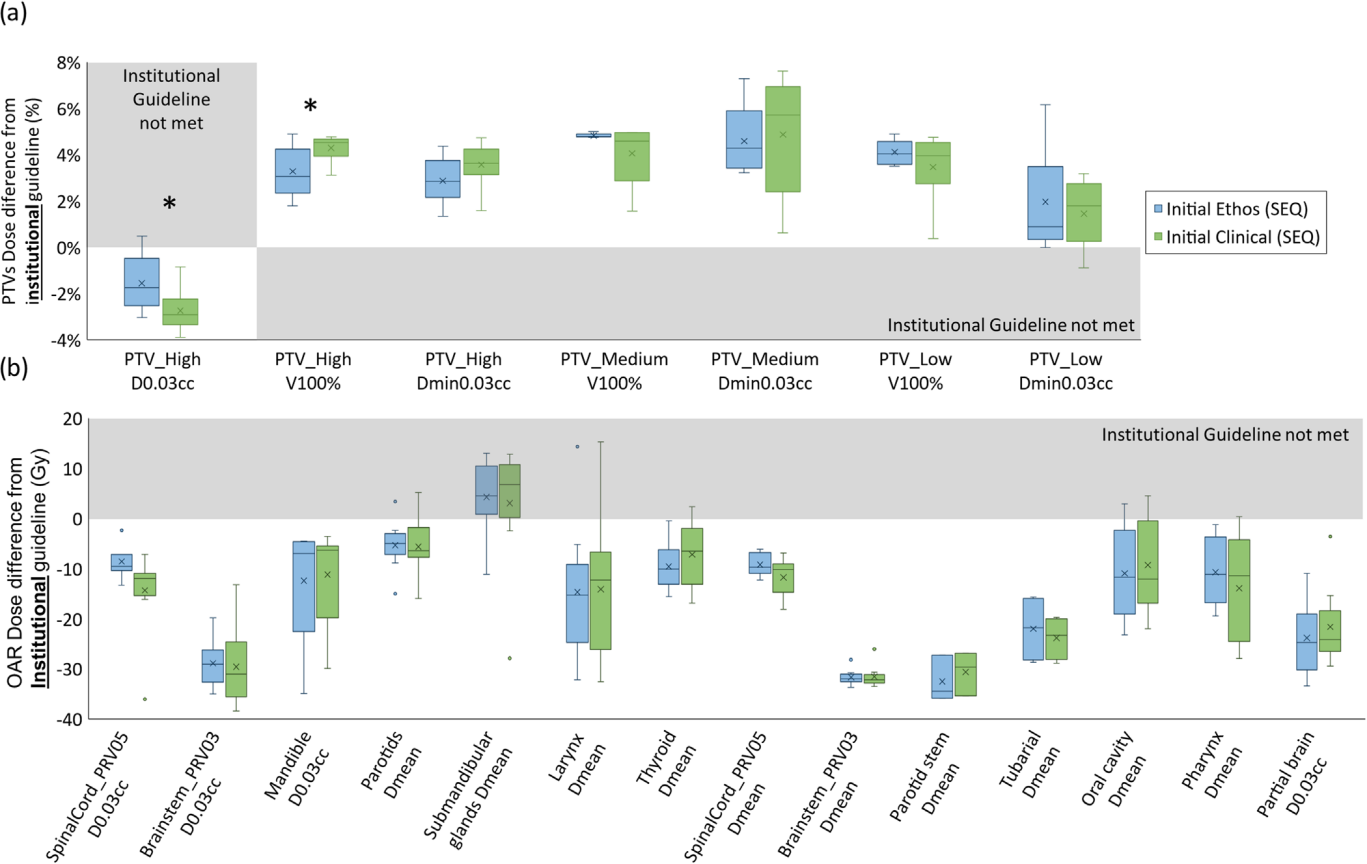
SEQ initial plan dosimetric evaluation. Distributions of (a) PTV (b) OAR dose differences between achieved values and institutional guidelines for Ethos (blue) and clinical (green) SEQ composite (base + boost) initial plans. The shaded areas indicate that a goal was not met. *Statistically significant difference (*p* < 0.05).

Figure 3 shows (a) PTV and (b) OAR dose differences from institutional guidelines for Ethos and clinical initial SEQ plans. Ethos initial SEQ plans were high quality with comparable PTV coverage (higher for some goals) with acceptable hotspots (average D_0.03cc_ 105.5% versus 104.3% in Ethos versus clinical plans). The use of the normal tissue high dose helper structure (Table 2) was found necessary to ensure that the highest dose (global hotspot) was inside the PTV. This was verified by ensuring that D_0.03cc_ was higher for PTV_High_ than normal tissue (Body—PTVs) in all plans except for one plan with a negligibly higher hotspot (6.6 cGy for the entire course) outside of the PTV_High_, with average achieved normal tissue D_0.03cc_ of 104.5%. Both Ethos and clinical initial SEQ plans similarly spared OARs, except for spinal cord PRV where the average dose was significantly higher in Ethos plans (D_0.03cc_ = 42 Gy vs. 36 Gy in clinical plans) but still below the guideline (50 Gy). The difference was statistically significant (favorable for clinical versus Ethos initial SEQ plans) for PTV_High_ D_0.03cc_ (*p* = 0.03) and PTV_High_ V_100%_ (*p* = 0.02).

The composite PQM statistics are shown in Table 3 for the Ethos/clinical‐initial SIB/SEQ plans. Higher PQM signifies higher target coverage and better OAR sparing. Both Ethos‐initial and clinical‐initial SIB and SEQ plans had comparable PQM averages (slightly higher in clinical plans due to specific dose metrics illustrated in Figure 2 and Figure 3, with no statistically significant differences).

### 3.2 Online adapted plans

Online adapted plans were generated by simulating the online adaptive workflow with physicist and physician participation for a single session in the Ethos emulator as described above for all 30 plans. Adapted plans were approved over scheduled plans for 29 of 30 instances. One scheduled base plan was selected to avoid undesired hotspot placement (in the mucosa) in the adapted plan at the cost of reduced target conformality (i.e., higher OAR doses).

**FIGURE 4:**
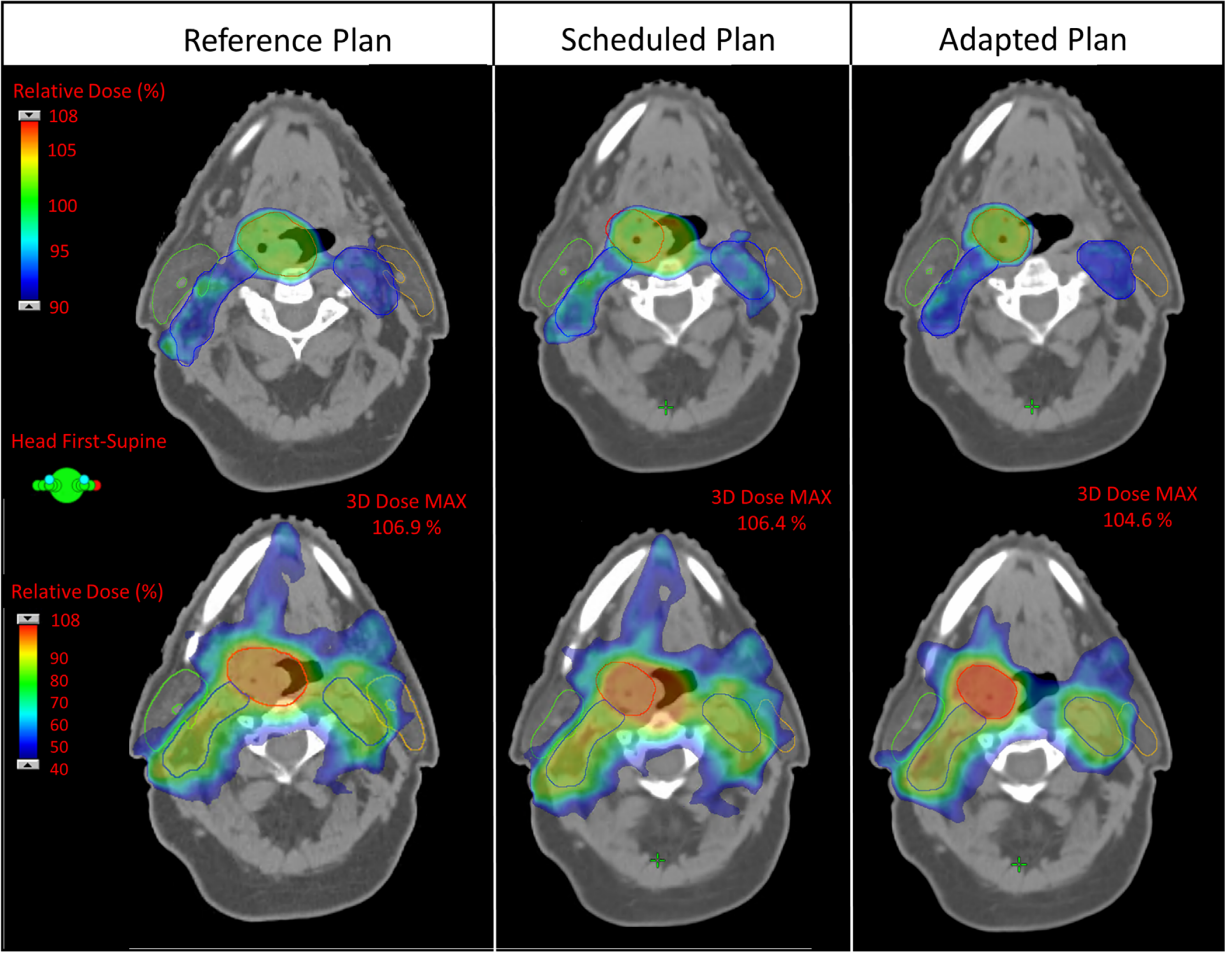
Dose color wash of the reference, scheduled and adapted Ethos plans for a representative SEQ base plan. The top and bottom rows show different slices with different dose colorwash ranges highlighting target coverage and OAR doses (e.g., parotid goal D_mean_ < 26 Gy here would be the 40% dose), respectively. Contours shown are: PTV_high_ (red), PTV_low_ (blue), right parotid (green), and left parotid (orange). Note, compared to the reference plan, the adapted contour for PTV_high_ (red) in columns 2 and 3 has decreased in size, resulting in over/under‐coverage and undesirable hotspots in the scheduled plan. Additionally, the scheduled plan shows the 40% dose spilling further into the left parotid. By comparison, the adapted plan provides conformality to the changed targets as well as better sparing of OARs and reduced heterogeneity (D_max_ < 105%).

Figure 4 shows the dose color wash of the SEQ base reference, scheduled and adapted plans for a representative patient. This example illustrates the benefits of online plan adaptation. Due to response and subsequent target shrinkage, the dose in the scheduled plan was no longer conformal to or adequately covering targets as in the original reference plan. This results in a suboptimal plan with unnecessary high dose spill into adjacent mucosa as well as medium dose (i.e., 40% of the PTV_High_ prescription dose, equivalent to 26 Gy) to nearby parotid glands that could potentially result in toxicity (e.g., xerostomia). By comparison, the adapted plan, reoptimized to the anatomy of the day with the clinical goals used to generate the reference plan, successfully rectifies these issues by maintaining desired target conformality, improved OAR avoidance, and improves homogeneity (D_max_ < 105%) to even be superior to that of the reference plan.

**FIGURE 5:**
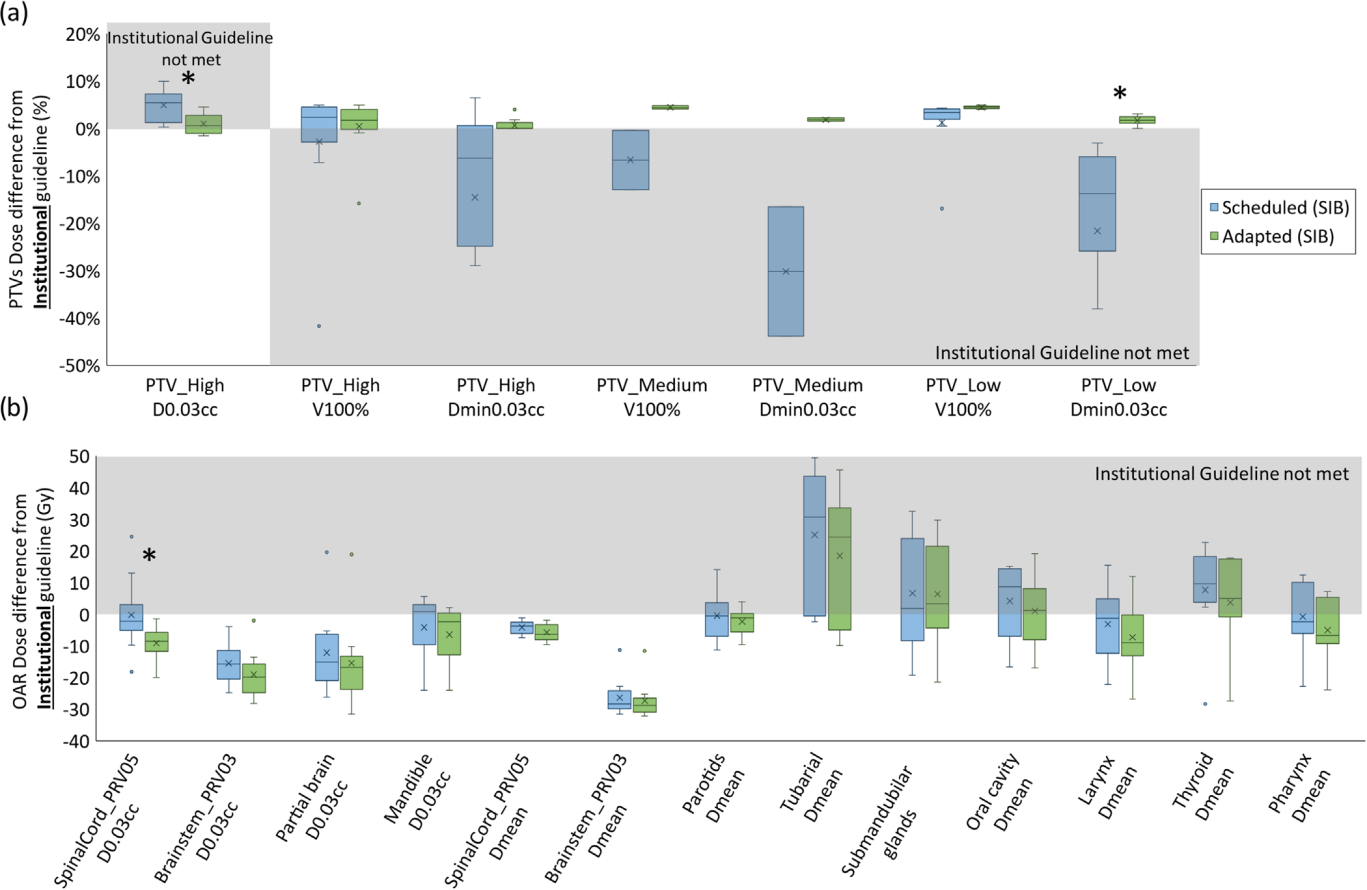
SIB adapted plan dosimetric evaluation. Distributions of (a) PTV and (b) OAR dose differences between achieved values and institutional guidelines for Ethos Scheduled (blue) and Ethos Adapted (green) SIB plans. The shaded areas indicate that a goal was not met. *Statistically significant difference (*p* < 0.05).

Figure 5 shows the dosimetric benefits of online adaptation with Ethos via (a) PTV and (b) OAR dose differences from institutional guidelines in Ethos‐scheduled and Ethos‐adapted SIB plans. Adaptation was quite useful as scheduled plans in many cases did not achieve PTV clinical goals, with PTV under‐coverage and high hotspots on average: PTV_High_ D_min,0.03cc_ = 80.6%/95.7%, PTV_Med_ D_min,0.03cc_ = 64.8%/96.9%, PTV_Low_ D_min,0.03cc_ = 73.4%/96.7%, and PTV High D_0.03cc_ = 111.9%/108.0% in Ethos‐scheduled/adapted plans, respectively. Normal tissue (Body—PTVs) average D_0.03cc_ was significantly higher in scheduled plans (111.4%) compared to adapted plans (106.5%) and was lower than the PTV_High_ D_0.03cc_ (i.e., global hotspot is in the PTVs) for 8/10 scheduled plans (average 35 cGy difference, for the full course, outside but proximal to PTV_High_) and 10/10 adapted plans. Overall, OAR doses trended higher in scheduled versus adapted plans, but only SpinalCord_PRV05 D_0.03cc_ was found to be statistically significant (*p* = 0.05). Overall, adapted SIB plans succeeded in achieving 100% of all PTV goals with superior OAR sparing compared to scheduled plans by an average improvement of 9% (range: 2−18%) lower dose across all specific OAR goals. In addition to inferior OAR sparing, scheduled SIB plans only achieved 14% of PTV goals across all plans.

**FIGURE 6:**
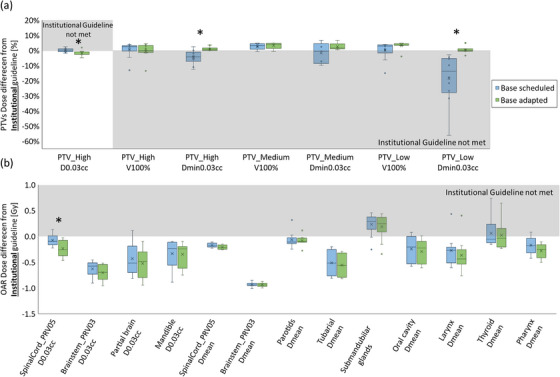
SEQ base phase adapted plan dosimetric evaluation. Distributions of (a) PTV (b) OAR dose differences between achieved values and institutional guidelines for Ethos Scheduled (blue) and Ethos Adapted (green) SEQ base plans. The shaded areas indicate that a goal was not met. *Statistically significant difference (*p* < 0.05).

**FIGURE 7:**
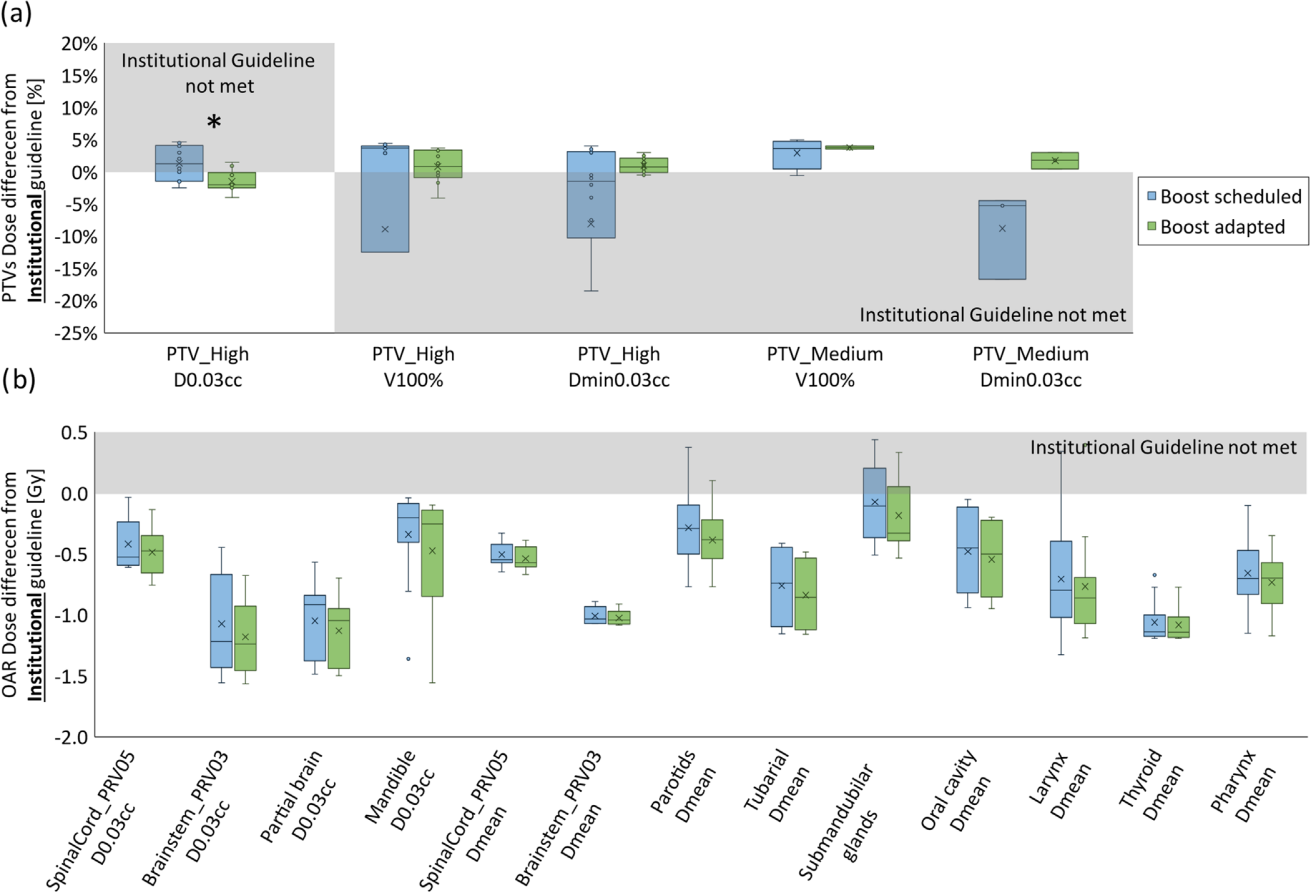
SEQ boost phase adapted plan dosimetric evaluation. Distributions of (a) PTV (b) OAR dose differences between achieved values and institutional guidelines for Ethos Scheduled (blue) and Ethos Adapted (green) SEQ boost plans. The shaded areas indicate that a goal was not met. *Statistically significant difference (*p* < 0.05).

Similar dosimetric benefits from online adaptation with Ethos in the SEQ plans are shown via dose difference from institutional guidelines for SEQ scheduled and adapted plans, which were evaluated separately for individual base and boost plans (Figure 6 and Figure 7, respectively), rather than for a composite. This represents how these plans would be evaluated and selected in a clinical online adaptive setting based on meeting goals independently. Adapted base and boost plans had higher PTV coverage while maintaining lower OAR doses and lower global hotspots, with the PTV_High_ D_0.03cc_ goal more consistently achieved in adapted plans. Adapted plans better spared normal tissue with D_0.03cc_ outside of PTVs of 107.5% versus 104.9% and 109.4% versus 104.2%, for scheduled versus adapted in base and boost plans, respectively. For some plans slightly higher hotspot was outside but proximal to PTV_High_ with improvement from scheduled to adapted plans of 40% to 80% in the base and 50% to 90% in the boost plans, respectively. The D_0.03cc_ outside of the PTV_High_ was marginally higher in these cases and decreased from the scheduled to adapted plans (from an average of 54.7 to 48.3 cGy and 60.0 to 6.0 cGy in the base and boost plans for the full phase, respectively). The difference between scheduled and adapted plans was statistically significant for PTV_High_ D_0.03cc_ (p_base_ = 0.01, p_boost_ = 0.01), PTV_High_ D_min,0.03cc_ (p_base_ = 0.01), PTV_Low_ D_min,0.03cc_ (p_base_ = 0.01), and SpinalCord_PRV05 D_0.03cc_ (p_base_ = 0.02). These statistically significant differences are indicated by asterisks in Figure 6 and Figure 7. Adapted SEQ plans succeeded in achieving 100% of PTV goals across all plans, whereas the scheduled plans only achieved 33% of these goals. Additionally, SEQ plans improved OAR sparing as assessed across all specific goals by an average of 10% (range: 2%−21%) lower dose versus scheduled plans.

The average composite PQM for Ethos‐ and clinical‐adapted SIB and SEQ (boost only) plans are shown in Table 3. All adapted plans had higher average composite PQM compared to the initial plans. The average composite PQM for both initial and adapted Ethos plans was comparable (no significant difference) but trended slightly lower compared to clinical plans. Only adapted boost plans for SEQ patients were compared here because most patients in this dataset only had boost plans adapted clinically.

**FIGURE 8:**
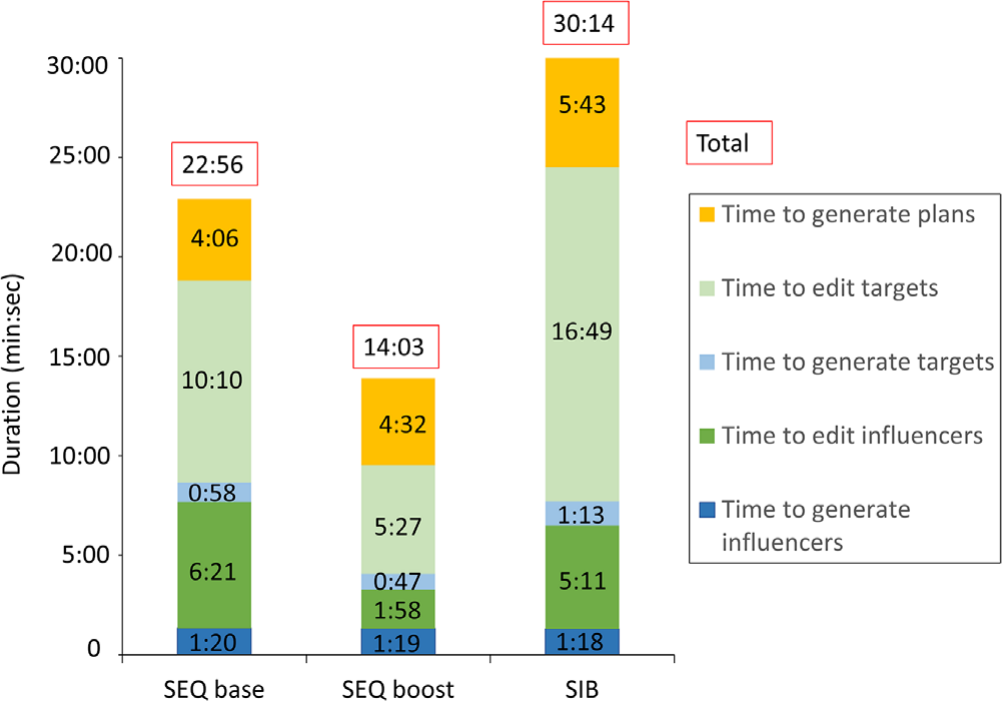
Duration (min: sec) of each component of the oART process for SEQ base, boost, and SIB plans, which were included in the emulator‐based workflow.

Timing data was collected for the five components of the adaptive workflow simulated within the emulator software, from influencer generation to plan generation (Figure 8). The average total duration for SEQ boost, SEQ base and SIB plans was 14:02, 22:56, and 30:14 (min:sec), respectively. Time to generate influencers and targets was the shortest and most consistent for all plans (on average 1:19 and 1:00, respectively). The duration of editing influencers was consistent (5:14) for SEQ base and SIB plans and was longer compared to SEQ boost plans (1:58). However, there was no difference in the influencers that were being edited for these three plan types. Therefore, this discrepancy was due to the physician learning curve and comfort level increasing between SIB/SEQ base plan adaptative sessions, which were performed first and SEQ boost plan adaptative sessions, which were performed last. The improvement in the duration to edit influencers by 3:36 was due to the user's experience. Duration to edit targets varied as a function of increasing target complexity for SEQ boost, SEQ base and SIB plans (on average 5:27, 10:10 and 16:49, respectively). The duration for plan generation for SIB and SEQ boost include both fixed field IMRT and full arc VMAT plans and was on average 5:43 and 4:32, respectively. However, full arc VMAT plan generation was longer than that of fixed field IMRT, with an average of 11:55 compared to 3:59 in the IMRT plans.

## 4 DISCUSSION

The Ethos, CBCT‐guided ring‐gantry oART system provides a platform with semi‐automated planning, re‐contouring and re‐planning for efficient adaptation of the treatment plan to the anatomy of the day. In this study, rather than investigating daily adaptation, or adaptation with a pre‐defined temporal sampling (e.g., weekly, every *n*‐fractions, beginning/middle/end of treatment, etc.), as has been done in previous studies (e.g.,^20^), we considered the extreme scenario with H&N patients known to clinically require offline adaptation due to appreciable changes in anatomy. This allowed us to evaluate the limits of Ethos for online adaptive H&N treatments with the expectation that actual average daily variations in patient anatomy will be less dramatic. Therefore, the promising results seen in this work would imply Ethos could successfully be used for oART in H&N for most clinical scenarios. In this work, an initial treatment planning approach for H&N cancer, using the new clinical goal planning paradigm that is robust to plan adaptation was developed. An Ethos emulator was utilized for initial planning and oART simulations in silico.

Different planning approaches were developed and tested on more complex SIB plans. Our first planning strategy, simply using all institutional guidelines as direct input for the Ethos clinical goals and/or excluding a subset of goals for OARs intersecting PTVs, failed to achieve acceptable initial plans. Our subsequent strategy, incorporating cropping of OARs intersecting PTVs (rather than relying on IOE auto‐cropping) and using minimal goals per OAR was found to be superior. All presented dosimetric results were based on using this approach with additional anatomically derived helper structures and goals for dose shaping (see Figure 1 and Table 2) and PTV air density override (if deemed reasonable and necessary). Similar density overrides are used in our clinic for standard H&N patient plans with the justification being: if gross disease moves within the uncertainty margin represented by the PTV to a region containing air in the simulation‐CT, the air at this location will have been replaced by tissue. The generated plans were more robust to variations in electron density abutting gross disease and within PTV margins. Ethos v1.1 does not allow importing density overridden structures, requiring manual delineation in an Ethos module (technical structures) separate from the target/OAR structure contouring, where it was not possible to see targets while drawing the overridden density structure. Furthermore, overridden density structures were not visible/editable during the adaptive workflow, instead a deformable propagation of the structures is performed. Future Ethos versions more robustly integrating technical structures during initial planning and inclusion of tools for density map verification/correction during online adaptation would be beneficial.

In this study, clinical plans were calculated based on collapsed cone convolution superposition, whereas Ethos used the more precise Acuros XB. Collapsed cone versus Monte Carlo (comparable to the Acuros XB) calculations of identical plans showed increased hot spots for Monte Carlo.^25^, ^27^, ^28^ For this reason, D_0.03cc_ ≤ 107% (vs. 105%) was used in this work. Ethos‐generated initial SIB plans struggled to meet the strict institutional guidelines for PTV homogeneity. Aside from this, most PTV coverage and OAR goals were clinically acceptable. For institutions using less stringent guidelines (e.g., those suggested in the cooperative group setting, such as NRG^29^) would encounter fewer issues. However, while the Ethos initial SIB plans struggled to achieve clinical acceptability, Ethos adapted SIB plans were more successful in achieving clinical goals with conformal/homogeneous target coverage while sparing OARs. This dosimetric improvement was most likely due to target size and complexity changes at the adaptive time point, which simplified plan optimization. Initial SEQ plans were comparable to clinical plans and in general met institutional guidelines. While superior manual plans could be achieved based on these comparisons, this approach requires more planning time/resources and would not allow for rapid online plan adaptation and superior daily dosimetry to changing anatomy. It is also worth noting that the capability to adjust plans to the anatomy of the day with oART decreases the uncertainties in daily anatomical variations that are accounted for traditionally with larger PTV margins. The Ethos plans in this work, used the same PTV margins as were used clinically without oART. In practice, these PTV margins can be confidently reduced when utilizing oART, as has been implemented in oART clinical trials.^30^

Fixed‐field IMRT plans were selected over VMAT plans 90.0% of the time in this work. This may change if future versions of Ethos enhance the VMAT optimizer. Fixed‐field IMRT plans were also favored in v1.1 due to faster optimization versus VMAT plans with an average plan generation of 3:59 versus 11:55. This acceleration in the adaptive workflow was also found in another study due to the faster fixed field IMRT optimization.^18^ Ethos generated highly modulated IMRT plans (average MUs per prescribed fractional dose of 6.8 ± 2.1 MU/cGy for all plans). However, this is not expected to be an issue since similarly modulated Ethos plans have been observed and validated for delivery accuracy in previous studies.^18^, ^31^

The institutional guidelines used for Ethos plan generation and evaluation in this work facilitated comparison with clinical plans but may make the results slightly less generalizable for other institutions. However, the institutional guidelines were mostly consistent with the established cooperative group metric NRG HN‐005 guidelines and, where they differed, were consistently more conservative.^29^ Use of the established PQM for plan evaluation helped to further ensure generalizability of the results. The average composite PQM represented overall achieved plan quality using objective scoring rather than subjective plan quality evaluation. For SEQ initial plans, individual Ethos plan metrics were superior compared to those for the clinical plans in many instances (Figure 3). However, the average composite PQM was higher in clinical plans. This was mostly due to the strict score function where, for example, a score of 0.0 would be assigned for PTV High V_100%_ = 94.9% (goal of 95.0%). It should also be noted that, due to the fact that patients selected for this work were known to have anatomical changes during treatment that were significant enough to require offline adaptation, it is an expected result that Ethos adapted plan quality will be superior to Ethos scheduled plan quality. This is still shown explicitly in Figures 5, 6, 7 not only to highlight that the Ethos adapted plans do well to achieve clinical goals in the face of such appreciable anatomic changes, but also to illustrate, via direct comparison to the scheduled plan results in these figures, the potential dosimetric manifestation of these anatomic changes that are avoided with online adaptation.

Influencers for all three plan types were the same, namely, mandible, parotids, spinal canal, and brainstem. The difference in time to edit influencers (Figure 8) was most likely a function of physician experience and the order that oART was simulated for these plans. The average of all influencer editing times, for all three plan types, may be more representative of an average experienced user. Auto‐generated influencers and targets were manually edited by physicians in all cases with average durations of 4:30 and 10:49 (min: sec), respectively. The incidence of manual edits and the amount of time required may represent an upper limit as this work presented the extreme situation where anatomical variation was known to be sufficient to have clinically required offline adaptation. Note, the emulator computer hardware was not identical to that of the clinical system and therefore the time to generate structures and plans reported herein may be diminished.

Patient setup, CBCT image acquisition, plan QA and beam delivery timing were not included in this emulator work. Median online adaptive duration after CBCT acquisition to plan selection was 18:56 (min: sec) and was similar to what Yoon et al. found (19:34). Missing steps were quantified by Yoon et al. to be image acquisition (1.6 min), QA (2‐3 min), and treatment delivery (1–2 min).^20^ Further improvements in the IOE/PO, auto‐contouring and the contouring tools will further accelerate this online adaptive process from end‐to‐end.

A limitation of this work was the use of offline adaptive simulation CT scans as pseudo‐CBCTs, which do not incorporate uncertainties associated with degraded imaging quality between fan‐beam CT and CBCT. This could include first‐order effects such as decreased contouring accuracy. This may also include second‐order effects, as the use of the offline adaptive simulation CT with higher quality and more consistent image intensities with the initial simulation CT (compared to the CBCT, which will be used in a real clinical scenario) in the adaptive workflow affects the DIR results. This would in turn affect generation of the synthetic CT and auto‐generated structures as well as dose calculation. This may diminish the accuracy of some results presented in this work compared to what will be observed in the true clinical scenario. However, advances in CBCT acquisition/reconstruction techniques now provide image quality approaching that of fan‐beam CT.^32^ Manual contour editing was found to almost always be necessary, as was found in other studies.^20^ Hence, auto‐contouring improvements could reduce oART treatment duration by up to ∼15 min (∼4.5 min for influencers and ∼11 min for targets). Another study stratified time to manually edit contours based on the quality of the auto‐contoured starting point for H&N oART with Ethos and found a decrease of treatment duration by ∼10 min (∼5 and ∼4.5 min for influencers and targets, respectively) when initial auto‐contours were high quality, which may represent a more realistic potential time savings.^20^ During oART, only underived targets (e.g., GTV and/or CTV_Med/Low_ used to derive PTV) were editable. Clinical CTVs derived from GTVs were routinely edited manually to avoid areas with no microscopic disease spread (e.g., air, bone, fascia or muscle). This was not possible in Ethos, where a consistent 5 mm margin around the GTV was maintained, which led to differences between Ethos and clinical‐adapted CTVs/PTVs.

Initial plan normalization was used for 75% of the plans to achieve the PTV_High_ D_min,0.03cc_ goal. However, within Ethos version 1.1 this normalization must be set prior to plan generation and will be applied to all online adapted plans. This might result in unwanted adapted plan degradation, especially in the extremely homogeneous H&N plans where a very small normalization change could produce a dramatic difference in target coverage. Consequently, avoiding normalization is preferable. In future versions, tools during oART to adjust normalization as a final step will be useful.

## 5 CONCLUSION

This study developed an initial planning strategy for H&N cancer with the new Ethos online adaptive system which, when compared to clinical plans generated with traditional planning approaches, resulted in acceptable plans with slightly lower quality for Ethos‐initial SIB plans and comparable quality for Ethos‐initial SEQ plans. In the current version, fixed‐field IMRT plans (versus VMAT plans) were found to be superior in quality and in optimization efficiency for the online adaptive workflow. Online adaptive sessions were successfully simulated in a time‐efficient manner for all plans, with adapted plans approved over scheduled plans in 97% of cases for achieving all PTVs goals (while scheduled plans would only have met 33%) with improvement in OAR sparing. These successful dosimetric results when using standard PTV margins suggest a promising potential to improve personalized treatments for patients in the future by implementing reduced margins as enabled by the reduced uncertainty associated with online adaptation.

Ethos oART allows for daily adaptation incorporating small changes in patient anatomy. This work tested the capabilities and robustness of this system by considering the extreme scenario where anatomical changes were significant enough to have clinically required offline adaptation. The success of Ethos adapted plans in adjusting the dose to these major changes in anatomy will ensure success of the daily online adaptation in most expected clinical scenarios.

This AI‐based online adaptive platform is a promising and powerful tool that will allow the generation of semi‐automated initial plans with the option of daily adaptation to ensure accurate and efficient treatment delivery by adjusting the treatment plan to changing patient anatomy. This is the first version of this novel system and continued development, particularly in VMAT optimization, automated contouring and CBCT image quality, will help facilitate more personalized daily treatments for H&N cancer patients.

## AUTHOR CONTRIBUTIONS

All of the above listed authors contributed directly to the intellectual content of the paper including work design and acquisition of data, writing/editing the manuscript, and final approval of this version.

## CONFLICT OF INTEREST STATEMENT

This work was funded by Varian Medical Systems, Palo Alto, CA.

## Supporting information

Supporting information

Supporting information
