# Supplementary figures and images for: A head and neck treatment planning strategy for a CBCT‐guided ring‐gantry online adaptive radiotherapy system
Source: J Appl Clin Med Phys. 2023 Aug 24;24(12):e14134. doi: 10.1002/acm2.14134 (PMC10691641; doi:10.1002/acm2.14134)

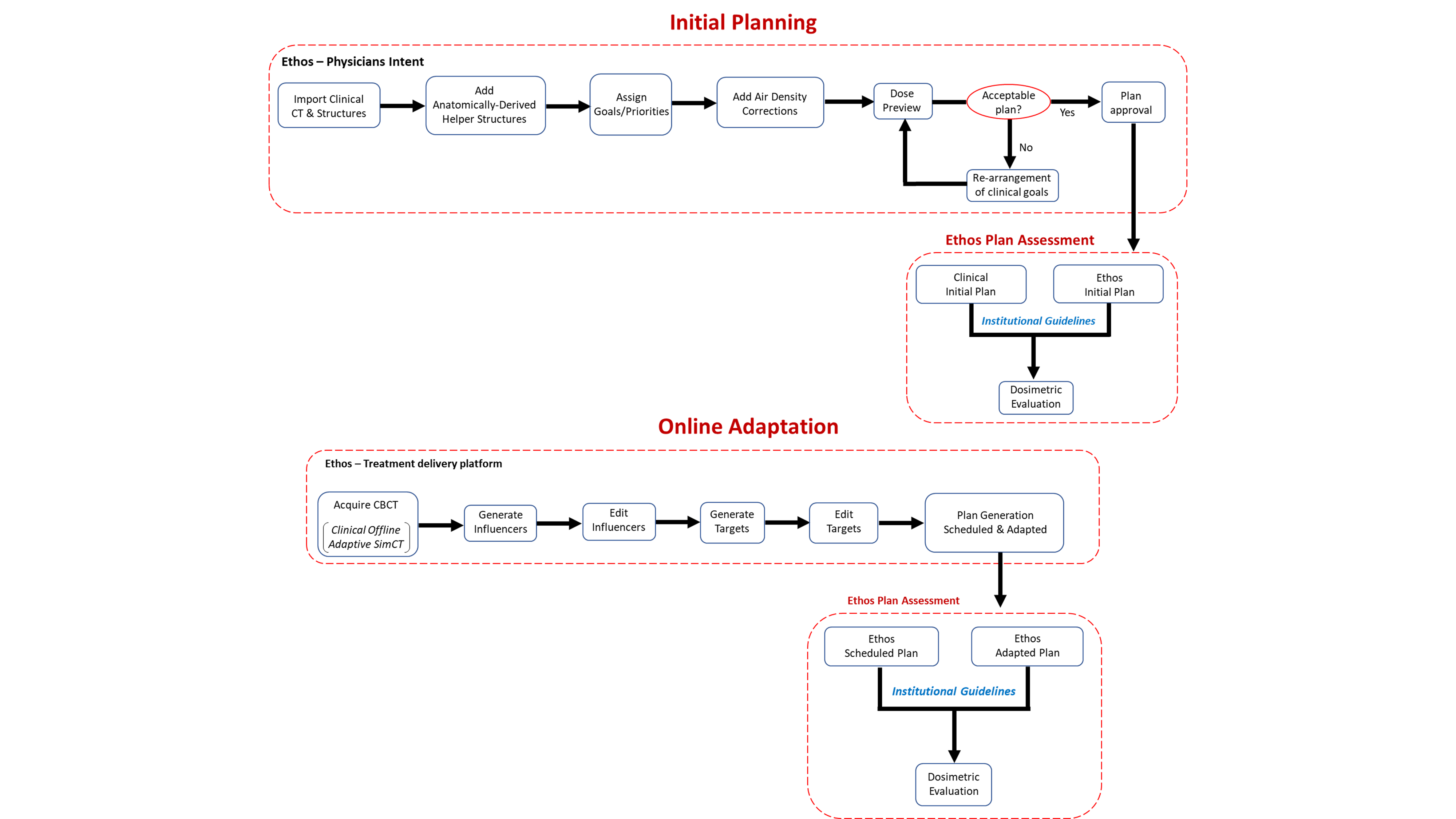

Supplement: Supplementary file 2 — Supporting information [file ACM2-24-e14134-s002.tif]
